# Continuing education as a strategy of public health surveillance for sustainable development

**DOI:** 10.1590/0034-7167-2024-0365

**Published:** 2025-11-17

**Authors:** Talita Lima do Nascimento, Herleis Maria de Almeida Chagas, Maria Francisca de Souza Rodrigues, Suleima Pedroza Vasconcelos

**Affiliations:** I Universidade Federal do Acre. Rio Branco, Acre, Brazil; II Universidade Federal do Amazonas. Manaus, Amazonas, Brazil

**Keywords:** Education, Continuing, Sustainable Development, Public Health Surveillance, Chronic Disease Indicators, Health Planning., Educación Continua, Desarrollo Sostenible, Vigilancia en Salud Publica, Indicadores de Enfermedades Crónicas, Planificación en Salud.

## Abstract

**Objectives:**

to describe the characteristics of participants and intervention projects from a continuing education course aimed at disseminating the 2030 Agenda for Sustainable Development in smaller, remote municipalities, aligned with the surveillance of chronic conditions and noncommunicable diseases.

**Methods:**

this is a descriptive, quantitative study conducted in the Brazilian states of Acre, Amazonas, Rondônia, and Roraima. Participants were health professionals affiliated with municipal and state public health surveillance systems. Data were collected from documents produced during a training course. Descriptive statistics were used for data analysis.

**Results:**

a total of 93 professionals participated, 85.7% of whom were women; 29.8% were between 26 and 36 years old; 73.4% held higher education degrees, and 46 were nursing professionals.

**Conclusions:**

the course was completed by 41 participants, who implemented intervention projects aligned with SDG 3.

## RESUMEN

**Objetivos:**

describir las características de los participantes y los proyectos de intervención de un curso de educación continua orientado a la difusión de la Agenda 2030 de los Objetivos de Desarrollo Sostenible en municipios remotos, en convergencia con la vigilancia de condiciones crónicas y enfermedades no transmisibles.

**Métodos:**

estudio descriptivo, con enfoque cuantitativo, realizado en los estados brasileños de Acre, Amazonas, Rondônia y Roraima. Los participantes fueron profesionales de la salud vinculados a las vigilancias municipales y estatales. La recolección de datos se basó en documentos producidos durante el curso. El análisis se realizó mediante estadística descriptiva.

**Resultados:**

participaron 93 profesionales; el 85,7 % eran mujeres, el 29,8 % tenía entre 26 y 36 años, el 73,4 % poseía formación superior y 46 eran profesionales de enfermería.

**Conclusiones:**

el curso fue finalizado por 41 participantes, quienes implementaron proyectos alineados con el ODS 3.

## INTRODUCTION

The Sustainable Development Goals (SDGs) were established by the United Nations (UN) as a bold strategy to promote dignified living conditions and eradicate global poverty. The 17 SDGs are outlined in a plan known as the 2030 Agenda, which includes indicators to be achieved and monitored by the signatory countries, including Brazil^(1)^.

Each goal includes a set of specific targets. SDGs 3, 5, 6, 8, and 16 align with the objectives defined in the *Strategic Action Plan for Addressing Noncommunicable Diseases and Related Conditions in Brazil, 2021-2030* (*Plano de Dant*). This plan reflects Brazil’s morbidity and mortality profile and indicates that 58% of all deaths in the general population are caused by noncommunicable diseases (NCDs)^(2)^.

“NCDs share a set of socioeconomic determinants and modifiable risk factors (RFs), which allow for a population-level intervention approach and public policies for their prevention and control”^(3)^. Through the Secretariat of Health and Environmental Surveillance, the Brazilian Ministry of Health incorporated this perspective when planning actions to expand the SDGs to smaller, remote areas of the country, strengthen their implementation at the municipal level, and support the achievement of the indicators established in the *Plano de Dant*.

Promoting equity and reducing injustices remain major challenges within the Brazilian health system. Considering the role of professionals in the health context, their proactive involvement in designing comprehensive and systematic plans that address the social determinants outlined in the SDGs is essential to achieving the targets proposed in both the 2030 Agenda and the *Plano de Dant* ^(4,5)^.

Implementing continuing education strategies is essential to build professional capacity focused on generating outcomes that address the current epidemiological profile of NCDs. “The training and development of health professionals is a practice embedded in healthcare work processes and environments, enabling opportunities for action and reflection on professional practices”^(6)^. Training developed within and for the workplace fosters the development of skills and the mobilization of knowledge that support interventions capable of producing change at the local level.

This study analyzes a course offered as part of an outreach project led by the Federal University of Acre in partnership with the Ministry of Health and three other universities in Northern Brazil: the Federal University of Amazonas, the Federal University of Rondônia, and the Federal University of Roraima. The aim was to develop and implement a health training strategy encompassing the 2030 Agenda and its convergence with the *Plano de Dant*. The structure of the training pathway, the profile of public health surveillance professionals, the implementation of the course, and its main outcomes are presented in this article.

The SDG framework is intrinsically connected to nursing practice^(5)^. Describing and linking nursing team practices to strategies grounded in sustainable development is essential for disseminating knowledge about actions that can influence professional practice and foster changes that contribute to enhancing health services.

## OBJECTIVES

To describe the characteristics of participants and intervention projects from a continuing education course aimed at disseminating the 2030 Agenda for Sustainable Development in smaller, remote municipalities, aligned with the surveillance of chronic conditions and noncommunicable diseases.

## METHODS

### Ethical aspects

This study was approved by the Research Ethics Committee (REC) of the Federal University of Acre in accordance with all guidelines established by the Brazilian National Health Council under Resolutions No. 466/2012 and No. 510/2016. Following approval from the RECs of the Federal Universities of Acre, Amazonas, Rondônia, and Roraima, we presented the study objectives and procedures to all participants. They subsequently signed an Informed Consent Form (ICF) indicating their participation agreement.

### Study design, period, and location

This is a multicenter descriptive study involving document analysis and a quantitative approach. The reporting followed the recommendations of the Strengthening the Reporting of Observational Studies in Epidemiology (STROBE) guidelines^(7)^.

Following the dissemination of the outreach project and coordination through the Bipartite Intermanager Committees in each state, we included all municipalities belonging to the health regions surrounding the capitals of the four states in the Western Amazon-Rio Branco (Acre), Manaus (Amazonas), Porto Velho (Rondônia), and Boa Vista (Roraima)-that expressed interest in the training process. The participating municipalities were:

- Acre: Acrelândia, Rio Branco, Plácido de Castro, Porto Acre, Bujari, Senador Guiomard, and Sena Madureira;
- Amazonas: Manaus, Iranduba, Presidente Figueiredo, Rio Preto da Eva, Manaquiri, Careiro da Várzea, and Barcelos;
- Rondônia: Porto Velho, Nova Mamoré, Itapuã do Oeste, Guajará-Mirim, and Candeias do Jamari;
- Roraima: Boa Vista, Cantá, Pacaraima, Mucajaí, Normandia, Rorainópolis, São Luís, and Uiramutã.

The course was delivered in two phases: a 16-hour in-person phase and a 64-hour distance education (DE) phase. During both phases, the outreach project coordination team applied two instruments: a participant profile questionnaire and an assessment tool for evaluating the design and implementation of the intervention projects.

The in-person phase was conducted in the state capitals between February and May 2024, and the distance education phase took place between May 2023 and January 2024.

### Population and inclusion criteria

All course participants-health professionals appointed by their respective municipalities and involved in the outreach project-were eligible for inclusion in the study sample. No exclusion criteria were established.

### Study protocol

Data collection was based on documents produced during the outreach project. In the in person phase, the coordinator and tutor administered a structured questionnaire to characterize participants in each state capital. In the distance education phase, a monitoring spreadsheet was used to track participant groups based on their municipality.

The participant profile questionnaire was applied on the first day of in-person training, prior to the start of educational activities. The variables collected included: gender, race/ethnicity, educational attainment, length of service in the health sector, time in current position, current job title, type of employment contract, academic training in the health field, and a description of that training.

The tutors completed the monitoring spreadsheet (assessment rubric) to monitor and evaluate project development. Participant groups were organized according to their municipality of origin to design and implement intervention projects (IPs). The rubric included six evaluation domains: project structure completion, written content, knowledge, feasibility, intended impact, and innovation. Each domain comprised a set of criteria used to classify the IP development stage as: completed, advanced development, early-stage development, or no development. Only IPs classified as “completed” were considered finalized. Those rated as “advanced development” or “early-stage development” were considered to be in partial implementation/in progress.

### Data analysis and statistics

Data were analyzed using descriptive statistics. Absolute and relative frequencies were calculated for categorical variables, and means were presented for continuous variables, all using Jamovi, a free and open-source statistical software package.

## RESULTS

Data on the characteristics of the professionals were collected using an electronic questionnaire administered on the first day of the in-person phase of the training course. Of the 93 enrolled participants, 84 (89.3%) adequately completed the sociodemographic and work-related information. Among these (n = 84), 72 (85.7%) were women, and 25 (29.8%) were between 26 and 36 years old. Regarding race/ethnicity, 60 (71.4%) self-identified as mixed race, and 3 (3.6%) as Indigenous. In terms of educational attainment, 60 (73.4%) held higher education degrees (Table 1).

**Table 1:** Distribution of sociodemographic and work-related variables among participants in the Sustainable Development Goals Outreach Project, 2024 (N = 84)

Variável	n	(%)
Gender		
Male	12	14.3
Female	72	85.7
Age group		
18-25 years	07	8.3
26-33 years	25	29.8
34-42 years	24	28.6
43-50 years	15	19.7
Over 50 years	13	15.5
Race/ethnicity		
White	09	10.7
Black	09	10.7
Mixed race	60	71.4
Asian	03	3.6
Indigenous	03	3.6
Educational attainment		
High school diploma	13	15.4
Bachelor’s degree	62	73.8
Graduate degree	09	10.7
State		
Acre	22	26.2
Amazonas	24	28.6
Rondônia	21	25.0
Roraima	17	20.2
Course completion		
In-person + distance education	39	46.4
In-person only	45	53.6
Length of service in the health sector		
Less than 1 year	03	3.6
1-5 years	42	50.0
6-10 years	10	11.9
11-15 years	14	16.7
More than 15 years	15	17.9
Time in current position		
Less than 1 year	24	25.3
1-5 years	59	62.1
6-10 years	09	9.5
11-15 years	01	1.1
More than 15 years	02	2.1
Training in the health field		
Yes	74	88.1
No	10	11.9
Employment arrangement		
Permanent	38	45.2
Temporary	46	54.8

As for work-related variables, 42 participants (50%) had between one and five years of experience in the health sector; 83 (87.4%) had held their current position for five years or less; and 74 (88.1%) had academic training in the health field. Among those who reported training in the health field, 68 (91.9%) described the nature of that training. Notably, there was a high number of nursing professionals-40 nurses and 6 nursing technicians. Other participants included psychologists, pharmacists, biomedical professionals, technicians, and endemic disease control agents. Regarding employment arrangements, 46 (54.8%) reported having temporary contracts, including commissioned positions, short-term contracts, or employment through outsourced service providers (Table 1).

The in-person training was conducted in the capitals of four states: Acre (n = 27), Amazonas (n = 24), Rondônia (n = 21), and Roraima (n = 21). It was delivered to 93 health professionals working in various sectors, including Epidemiological and Health Surveillance, Primary Health Care, Endemic Disease Control, Nursing Coordination, and Health Planning. All professionals were appointed by municipal health managers from their respective municipalities. Of the 93 participants, 52 (55.9%) completed only the in-person phase (16 hours) and therefore did not complete the full course. A total of 41 participants (44.1%) completed the full course, which included the 64-hour distance education phase, totaling 80 hours of instruction. Among those who completed the course, 36 (87.8%) fully implemented their intervention projects, as shown in Chart 1.

**Chart 1:** Description and implementation status of intervention projects by municipality and state

Title of the IP	Related SDG	Municipality/state	Number of participants	IP status
Health Intervention Project on Mental Health in Primary Health Care in the Municipality of Senador Guiomard	SDG 3	Senador Guiomard/AC	1	Completed
Monitoring and follow-up of the prevalence of diabetes mellitus and hypertension at the Eduardo Francisco de Paiva Family Health Unit	SDG 3	Rio Branco/AC	6	Completed
Intervention project to increase adherence to hypertension treatment in Primary Health Care and to prevent cardiovascular diseases and conditions	SDG 3	Sena Madureira/AC	4	Completed
Intervention addressing the high prevalence of hypertension and diabetes in Plácido de Castro	SDG 3	Plácido de Castro/AC	3	Completed
Bujari says NO to sexual violence against children and adolescents!	SDG 3 and 5	Bujari/AC	3	In progress
Obesity/Overweight: Creating Healthy Habits	SDG 3	Manaquiri/AM	3	Completed
Stroke-Free Community	SDG 3	Manaus/AM	3	Completed
Hypertension in the workplace: risk factors, causes, effects, promotion, and prevention	SDG 3	Presidente Figueiredo/AM	2	Completed
Prevention and control of hypertension through nutrition - Senior Center	SDG 3	Rio Preto da Eva/AM	1	Completed
Vaccination is protection - Porto Velho in the fight against HPV	SDG 3	Porto Velho/RO	3	Completed
“Coffee and Conversation”: Perception and risk of mental health issues among public employees in the workplace	SDG 3	Porto Velho/RO	4	Completed
Hypertension: proposal for a control action plan under the Family Health Strategy, Planalto area, Nova Mamoré/RO	SDG 3	Nova Mamoré/RO	2	Completed
Live Well Itapuã - yes to health, no to diabetes and hypertension	SDG 3	Itapuã do Oeste/RO	2	Completed
Masculinity versus Health	SDG 3	Boa Vista/RR	1	In progress
Promoting good quality of life	SDG 3	Cantá/RR	1	Completed
Vital Activity: HIPERDIA in Motion!	SDG 3	Mucajai/RR	1	Completed
Reducing risk factors associated with the development of hypertension	SDG 3	Pacaraima/RR	1	In progress

During the distance education phase, the expected outcome was the development of one intervention project (IP) per participating municipality. A total of 17 IPs were submitted-5 from Acre, 4 from Amazonas, 4 from Rondônia, and 4 from Roraima-of which 14 (82.3%) were fully implemented. Notably, projects addressing hypertension and diabetes accounted for 52.9% of all submissions. A detailed description of the intervention projects by state is presented in Chart 1.

## DISCUSSION

To implement public health surveillance actions, “it is essential to consider the training of workers, which is a key factor in developing competencies and skills”^(8)^. Accordingly, it is crucial to offer courses and other forms of health education that promote competency development and equip professionals to advance health and achieve established targets^(8)^.

The connection between SDG 3 and the field of health surveillance is evident in the alignment of national targets. Brazil is an SDG signatory and has committed to the 2030 Agenda. To support the monitoring of these goals, the Ministry of Health established the Advisory Committee for Monitoring and Evaluation of the Unified Health System. Additionally, to reinforce this commitment, the Ministry revised the *Plano de Dant*, incorporating actions related to the SDGs and aligned with the 2030 Agenda. In this context, training initiatives that foster understanding of the Agenda and its integration with health surveillance are essential to engaging professionals in meeting the established goals^(9)^.

The course analyzed in this study was delivered in a distance education format. According to a study by Brasil et al. (2023), this is “a learning modality that provides flexibility in time and space, enabling broader participation in educational activities without compromising quality”. This format can expand access for professionals living in remote areas who face difficulties with travel and taking leave from work to attend in-person training sessions^(10)^.

The data on workers in this study align with findings from a survey conducted with Ministry of Health employees, in which 67.6% were women, 53.3% self-identified as white, 66.7% held graduate degrees, and 38.7% reported an income between five and ten minimum wages. A predominance of women was observed. In our study, most participants self-identified as mixed race, while in the referenced survey, most identified as white. However, it is important to note that our study focused on the Amazon region, where, according to the Brazilian Institute of Geography and Statistics (IBGE), the population is predominantly mixed race and Indigenous. In our findings, most participants had completed higher education, whereas in the study by Matielo et al. (2023), most health workers held graduate degrees. This difference likely reflects regional disparities in educational opportunities, as the North region has fewer graduate programs than other regions of the country^(11,12)^.

A study examining the professional profile of health surveillance workers in the state of Pernambuco identified considerable variation in the types of higher education professionals working in the field. These results are corroborated by our study, which identified psychologists, pharmacists, biomedical professionals, technicians, and endemic disease control agents working in health surveillance despite the predominance of nursing professionals^(13)^. Mendes et al. (2022) reported that nursing professionals represent approximately 70% of Brazil’s health workforce. However, there are regional disparities in its distribution: the Southeast has the highest concentration of these professionals, while the North and Northeast are most affected by unequal distribution”^(14)^.

Regarding the employment arrangements of course participants, the majority (54.8%) held temporary contracts, working in commissioned positions or under private-sector contracts through outsourced service providers for the health system. This employment structure may contribute to high turnover among health surveillance professionals, resulting in fragmented work processes and discontinuity of planned actions. Turnover-related issues in this sector were also described by Costa et al. (2023), who, in addition to the factors mentioned above, noted a sense of non-belonging among team members and political interference, which ultimately weakens surveillance activities, disrupts operational routines, and hinders team coordination, as professionals are constantly being replaced^(13,15)^.

During the course, 23 intervention projects were developed, of which 16 were fully completed. It is important to highlight that a situational diagnosis was conducted in each municipality beforehand, allowing participants to identify a health issue based on the local epidemiological profile and the *Plano de Dant* and subsequently propose an appropriate intervention. According to Gallo (2012), health education practices that take the territory and its dynamics into account are more effective^(16)^. Information and Communication Technologies in Health (ICTs in Health) were integrated into the distance education modality, helping overcome geographical barriers and enabling tutors and participants to receive timely academic support. Additionally, ICTs “increase interactivity between students and tutors and broaden the perspective for knowledge production”^(17)^. This support may have contributed to the implementation of 66.9% of the projects by the end of the course.

The most frequently addressed topic (52.9%) among the intervention projects was hypertension and diabetes. The selection of this theme aligns with findings from a study that examined changes in the prevalence and distribution of NCDs, demonstrating that social vulnerability, inequality, and poor economic conditions are associated with an increase in these conditions. Addressing these diseases is directly related to the work of nurses in primary health care and health surveillance and is also linked to the indicators and targets established in both the *Plano de Dant* and SDG 3^(3,18)^.

A study by Melo et al. (2019) identified a 56.7% prevalence of the following NCDs: hypertension, diabetes, and dyslipidemia. These findings were observed in a low-income population with limited educational attainment, similar to many municipalities in the Amazon region included in the present study. Most participants’ choice of NCDsmay have been influenced by their perception of a recent increase in the prevalence of these diseases in their local communities^(19)^.

Nursing holds significant potential to contribute actively to achieving the SDGs and implementing the 2030 Agenda, “beginning with greater awareness through education and a commitment to research and participation in local and global decision-making”^(20)^. The nursing workforce is fundamental to achieving SDG 3. However, leadership development, the strengthening of educational processes, and continued investment in research are requirements and ongoing challenges for nursing-particularly in Latin America-to advance its contributions toward achieving the SDGs^(21)^.

### Study limitations

Incomplete completion of forms by participants resulted in a reduced sample size.

### Contributions to the field of Nursing and Public Health

This study contributes by providing knowledge on continuing education initiatives related to sustainable development and its intersection with health-particularly in the area of surveillance of chronic conditions and noncommunicable diseases. It underscores the role of nursing professionals in leading the planning and implementation of health surveillance activities in municipalities of the Western Amazon region of Brazil.

## CONCLUSIONS

The course, offered as part of an outreach project addressing the SDGs, the 2030 Agenda, and its connection with the *Plano de Dant*, was fully completed by 44.08% of participants enrolled across the four states of the Western Amazon. A total of 17 intervention projects were developed, focusing on SDG 3 (Good Health and Well-being) and SDG 5 (Gender Equality), defined based on local priorities and the epidemiological profiles of the participating municipalities-particularly regarding hypertension and diabetes mellitus. The analysis of the educational backgrounds of health surveillance workers showed that 73.8% held higher education degrees, with nursing being the most representative professional category. These findings reinforce the importance of empowering nursing professionals and strengthening their leadership in implementing the 2030 Agenda.

## Data Availability

The research data are available within the article.
