# Supplementary material for: Continuing education as a strategy of public health surveillance for sustainable development
Source: Rev Bras Enferm. 2025 Nov 17;78(Suppl 3):e20240365. doi: 10.1590/0034-7167-2024-0365 (PMC12622973; doi:10.1590/0034-7167-2024-0365)
Supplement: Translation

# Translation: A educação continuada como estratégia da vigilância em saúde pública para o desenvolvimento sustentável

**DOI:** 10.1590/0034-7167-2024-0365pt

**Authors:** Talita Lima do Nascimento, Herleis Maria de Almeida Chagas, Maria Francisca de Souza Rodrigues, Suleima Pedroza Vasconcelos

**Affiliations:** I Universidade Federal do Acre. Rio Branco, Acre, Brasil; II Universidade Federal do Amazonas. Manaus, Amazonas, Brasil

**Keywords:** Educação Continuada, Desenvolvimento Sustentável, Vigilância em Saúde Pública, Indicadores de Doenças Crônicas, Planejamento em Saúde.

## Abstract

**Objetivos:**

descrever as características dos cursistas e dos projetos de intervenção de um curso de educação continuada voltado à disseminação da Agenda 2030 dos Objetivos de Desenvolvimento Sustentável em municípios do interior, em convergência com a vigilância de agravos e doenças crônicas não transmissíveis.

**Métodos:**

trata-se de estudo descritivo, de abordagem quantitativa, realizado nos estados do Acre, Amazonas, Rondônia e Roraima. Os participantes foram profissionais de saúde vinculados às Vigilâncias em Saúde municipais e estaduais. A coleta de dados deu-se por meio de documentos produzidos durante um curso de formação. A análise dos dados foi efetuada utilizando se estatística descritiva.

**Resultados:**

participaram 93 profissionais, dos quais 85,7% eram do sexo feminino, 29,8% estavam na faixa etária de 26 a 36 anos, 73,4% tinham ensino superior, e 46 eram profissionais de enfermagem.

**Conclusões:**

o curso foi concluído por 41 participantes, que implementaram projetos de intervenção alinhados com o ODS 3.

## INTRODUÇÃO

Os Objetivos de Desenvolvimento Sustentável (ODS) foram definidos pela Organização das nações Unidas (ONU) como uma estratégia ousada para promover condições dignas de vida às pessoas e erradicar a pobreza no mundo. Os 17 ODS estão descritos em um plano conhecido como a Agenda 2030, contendo indicadores que devem ser alcançados e monitorados pelos países signatários dessa agenda, incluindo o Brasil^(1)^.

Cada objetivo conta com um conjunto de metas a serem alcançadas. Os ODS 3, 5, 6, 8 e 16 convergem com as metas delineadas no Plano de Ações Estratégicas para o Enfrentamento das Doenças Crônicas e Agravos não Transmissíveis no Brasil, 2021-2030 (Plano de Dant). O plano considera o perfil de morbimortalidade brasileiro e aponta que 58% das causas de morte na população em geral são por doenças crônicas não transmissíveis (DCNT)^(2)^.

“As DCNT têm em comum um conjunto de determinantes socioeconômicos e fatores de risco (FR) modificáveis que resultam na possibilidade de se ter uma abordagem de intervenção populacional e de políticas públicas para sua prevenção e controle”^(3)^. Por meio da Secretaria de Vigilância em Saúde e Ambiente, o Ministério da Saúde considerou essa possibilidade ao planejar ações voltadas à expansão dos ODS para o interior do país (interiorização), fortalecendo sua implementação nos municípios e buscando o cumprimento dos indicadores estabelecidos no Plano de Dant.

Os desafios de promoção da equidade e redução de injustiças são uma realidade no sistema de saúde brasileiro. Considerando o papel dos profissionais dentro do contexto da saúde, sua atuação proativa na elaboração de planejamentos abrangentes e sistemáticos que abordem os determinantes sociais pautados pelos ODS é fundamental para o alcance das metas propostas na Agenda 2030 e no Plano de Dant^(4,5)^.

É imprescindível implementar estratégias de educação continuada para o processo de instrumentalização dos profissionais focado em produção de resultados que atendam a esse cenário epidemiológico das DCNT. “A formação e desenvolvimento de profissionais de saúde, é uma prática presente nos processos e ambientes de trabalho em saúde e possibilita espaços de ação e reflexão das práticas”^(6)^. Com base na formação no (e para o) trabalho, desenvolvem-se habilidades e mobilizam se conhecimentos que possibilitam a realização de intervenções capazes de produzir mudanças no cenário local.

Este estudo refere-se a um curso inserido num projeto de extensão desenvolvido pela Universidade Federal do Acre, em parceria com o Ministério da Saúde e mais três universidades do Norte do Brasil: Universidade Federal do Amazonas, Universidade Federal de Rondônia e Universidade Federal de Roraima. O intuito era desenvolver e implementar uma estratégia de formação em saúde que contemplasse a Agenda 2030 e sua convergência com Plano de Dant. O delineamento do percurso formativo, o perfil dos profissionais a área de vigilância e a execução da formação em si e seus principais resultados são apresentados neste trabalho.

A temática dos ODS está intrinsicamente relacionada com a atuação da enfermagem^(5)^. Descrever e associar as práticas das equipes de enfermagem com estratégias pautadas no desenvolvimento sustentável é essencial para a difusão de conhecimentos sobre ações capazes de impactar a prática profissional e promover mudanças que contribuem com os serviços de saúde.

## OBJETIVOS

Descrever as características dos cursistas e dos projetos de intervenção de um curso de educação continuada voltado à disseminação da Agenda 2030 dos Objetivos de Desenvolvimento Sustentável em municípios do interior, em convergência com a vigilância de agravos e doenças crônicas não transmissíveis.

## MÉTODOS

### Aspectos éticos

Este estudo foi aprovado pelo Comitê de Ética em Pesquisa (CEP) com Seres Humanos da Universidade Federal do Acre, seguindo todas as normas estabelecidas pelo Conselho Nacional de Saúde nas Resoluções nº 466/2012 e nº 510/2016. Após aprovação pelos CEPs das universidades federais do Acre, Amazonas, Rondônia e Roraima, apresentamos os objetivos e procedimentos da pesquisa aos participantes. Posteriormente, eles assinaram um Termo de Consentimento Livre e Esclarecido (TCLE) concordando em participar da pesquisa.

### Desenho, período e local do estudo

Trata-se de estudo multicêntrico descritivo com análise documental e abordagem quantitativa. Foram seguidas as recomendações do *Strengthening The Reporting of Observational Studies in Epidemiology* (STROBE)^(7)^.

Após a divulgação do projeto de extensão e pactuação nas Comissões Intergestores Bipartites de cada estado, foram atendidos todos os municípios que compunham as regiões de saúde das quatro capitais dos estados da Amazônia Ocidental (Rio Branco - Acre; Manaus - Amazonas; Porto Velho - Rondônia; e Boa Vista - Roraima) e que manifestaram interesse no processo de formação, sendo eles os seguintes:

- Acre: Acrelândia, Rio Branco, Plácido de Castro, Porto Acre, Bujari, Senador Guiomard e Sena Madureira;
- Amazonas: Manaus, Iranduba, Presidente Figueiredo, Rio Preto da Eva, Manaquiri, Careiro da Várzea e Barcelos;
- Rondônia: Porto Velho, Nova Mamoré, Itapuã do Oeste, Guajará Mirim e Candeias do Jamari;
- Roraima: Boa Vista, Cantá, Pacaraima, Mucajaí, Normandia, Rorainópolis, São Luís e Uiramutã.

O curso ocorreu em duas etapas: uma presencial com 16 horas e uma de ensino à distância (EaD) com 64 horas. Nessas etapas, a coordenação do projeto de extensão aplicou um instrumento de caracterização do perfil dos profissionais de saúde e um instrumento de avaliação da elaboração e execução dos projetos de intervenção.

A etapa presencial transcorreu nas capitais, entre fevereiro e maio de 2024; e a etapa à distância, entre maio de 2023 e janeiro de 2024.

### População e critérios de inclusão

Todos os participantes do curso, que eram profissionais de saúde indicados pelos seus respectivos municípios e integravam o projeto de extensão, foram elegíveis para compor a amostra deste estudo, não havendo critérios de exclusão previstos.

### Protocolo do estudo

A coleta de dados foi realizada por meio de documentos produzidos no projeto de extensão. Na etapa presencial, a coordenadora e a tutora aplicaram questionário estruturado de caracterização dos cursistas em cada capital. Na etapa EaD, preencheram uma planilha de monitoramento dos grupos de cursistas por município.

O questionário de caracterização dos cursistas foi aplicado no primeiro dia da formação presencial do curso, antes do início das atividades pedagógicas. As variáveis coletadas foram: gênero, raça/cor, nível de escolaridade, tempo de atuação no setor saúde, tempo de atuação no cargo atual, cargo ocupado, tipo de vínculo empregatício, formação na área da saúde e descrição da formação.

A planilha de monitoramento (rubrica de avaliação) foi um instrumento preenchido pelas tutoras com a finalidade de acompanhar e avaliar o desenvolvimento das atividades. Os grupos de participantes foram formados conforme o município, com o objetivo de elaborar e executar projetos de intervenção (PI). Essa rubrica foi aplicada pela tutoria contendo seis tópicos de avaliação: estrutura do projeto preenchida, produção textual, conhecimento, viabilidade, impacto pretendido e inovação. Cada tópico continha um conjunto de critérios que permitia classificar o estágio do PI como: desenvolvido, desenvolvimento avançado, desenvolvimento iniciado e sem desenvolvimento. Somente os PIs classificados como “desenvolvido” foram considerados concluídos. Aqueles avaliados como “desenvolvimento avançado” e “desenvolvimento iniciado” foram considerados parcialmente concluídos.

### Análise dos resultados e estatística

A análise dos dados foi realizada por meio de estatística descritiva. Apresentaram-se frequências absolutas e relativas para as variáveis nominais e médias para as variáveis contínuas, com auxílio do software Jamovi, um pacote estatístico de acesso livre.

## RESULTADOS

Os dados sobre as características dos profissionais foram coletados mediante um questionário eletrônico e aplicados no primeiro dia da etapa presencial do curso de formação. Dos 93 cursistas matriculados, 84 (89,3%) preencheram adequadamente as informações sociodemográficas e relacionadas ao trabalho. Dentre esses (n = 84), 72 (85,7%) eram do gênero feminino, 25 (29,8%) estavam na faixa etária de 26 a 36 anos. Quanto à raça/cor, 60 (71,4%) declararam-se pardos; e três (3,6%), indígenas. No tocante à escolaridade, 60 (73,4) tinham ensino superior completo (Tabela 1).

**Tabela 1:** Distribuição das variáveis sociodemográficas e relacionadas ao trabalho dos cursistas do Projeto de Extensão dos Objetivos de Desenvolvimento Sustentável, 2024 (N = 84)

Variável	n	(%)
Sexo		
Masculino	12	14,3
Feminino	72	85,7
Faixa etária		
De 18 a 25 anos	07	8,3
De 26 a 33 anos	25	29,8
De 34 a 42 anos	24	28,6
De 43 a 50 anos	15	19,7
> de 50 anos	13	15,5
Cor		
Branca	09	10,7
Preta	09	10,7
Parda	60	71,4
Amarela	03	3,6
Indígena	03	3,6
Escolaridade		
Ensino médio	13	15,4
Ensino superior	62	73,8
Pós-graduação	09	10,7
Estado		
Acre	22	26,2
Amazonas	24	28,6
Rondônia	21	25,0
Roraima	17	20,2
Conclusão da formação		
Presencial + EaD^*^	39	46,4
Presencial	45	53,6
Tempo de atuação no setor saúde		
Menos de 1 ano	03	3,6
De 1 a 5 anos	42	50,0
De 6 a 10 anos	10	11,9
De 11 a 15 anos	14	16,7
Mais de 15 anos	15	17,9
Tempo de atuação no cargo atual		
Menos de 1 ano	24	25,3
De 1 a 5 anos	59	62,1
De 6 a 10 anos	09	9,5
De 11 a 15 anos	01	1,1
Mais de 15 anos	02	2,1
Possui formação na área da saúde		
Sim	74	88,1
Não	10	11,9
Vínculo de trabalho		
Efetivo	38	45,2
Temporário	46	54,8

* *Ensino à distância.*

No que tange às variáveis relacionadas ao trabalho, 42 (50%) cursistas tinham entre um e cinco anos de atuação na área da saúde, 83 (87,4%) estavam no cargo atual por um período de cinco anos ou menos, e 74 (88,1%) tinham formação na área da saúde. Dentre os profissionais que indicaram formação na área da saúde, 68 (91,9%) descreveram a natureza da formação. Vale ressaltar a elevada participação dos profissionais de enfermagem, sendo 40 enfermeiros e 6 técnicos de enfermagem. Em menor número, havia psicólogos, farmacêuticos, biomédicos, técnicos e agentes de endemias. Quanto ao vínculo de trabalho, 46 (54,8%) referiram que os contratos eram temporários: cargos comissionados, contratos temporários e vinculação a empresas terceirizadas (Tabela 1).

A formação presencial foi realizada nas capitais de quatro estados: Acre (n = 27), Amazonas (n = 24), Rondônia (n = 21) e Roraima (n = 21). Foi ministrada a 93 profissionais de saúde que atuavam em diversos setores como Vigilância Epidemiológica e Sanitária, Atenção Primária à Saúde, Coordenação de Endemias, Coordenação de Enfermagem, bem como Planejamento. Todos os profissionais foram indicados pelos gestores municipais de saúde de cada município. Dos 93 cursistas, 52 (55,9%) realizaram apenas a etapa presencial, que correspondia a 16 horas, portanto não concluíram o curso. Um total de 41 cursistas (44,1%) concluiu o curso, ou seja, cumpriu também as 64 horas de educação à distância (EaD), totalizando uma carga horária de 80 horas. Dentre os que concluíram o curso, 36 (87,8%) executaram integralmente o projeto de intervenção, conforme especificado no Quadro 1.

**Quadro 1:** Descrição e status dos projetos de intervenção dos municípios por estado

Título do PI	ODS Relacionado	Município/estado	N de participantes	Fase do PI
Projeto de Intervenção em Saúde Mental na Atenção Primária à Saúde do Município de Senador Guiomard	ODS 3	Senador Guiomard/AC	1	Concluído
Monitoramento e acompanhamento da prevalência de diabetes *mellitus* e hipertensão arterial sistêmica na Unidade de Saúde da Família Eduardo Francisco de Paiva	ODS 3	Rio Branco/AC	6	Concluído
Projeto de intervenção para aumentar a Adesão ao Tratamento de Hipertensos na Atenção Primária, e Prevenção de Doenças e Agravos Cardiovasculares	ODS 3	Sena Madureira/AC	4	Concluído
Intervenção na alta prevalência de hipertensos e diabéticos em Plácido de Castro	ODS 3	Plácido de Castro/AC	3	Concluído
Bujari diz NÃO à violência sexual contra crianças e adolescentes!	ODS 3 e 5	Bujari/AC	3	Parcialmente concluído
Obesidade/Sobrepeso: Criando Hábitos Saudáveis	ODS 3	Manaquiri/AM	3	Concluído
Comunidade sem Acidente Vascular Cerebral	ODS 3	Manaus/AM	3	Concluído
Hipertensão arterial no ambiente de trabalho: fatores de risco, causas, efeitos, promoção e prevenção	ODS 3	Presidente Figueiredo/AM	2	Concluído
Prevenção e combate à hipertensão arterial por meio da alimentação - centro do idoso	ODS 3	Rio Preto da Eva/AM	1	Concluído
Vacinar é proteger - Porto Velho na luta contra o HPV	ODS 3	Porto Velho/RO	3	Concluído
“Café com Prosa”: Percepção e risco à Saúde mental de Servidores Públicos no âmbito de trabalho	ODS 3	Porto Velho/RO	4	Concluído
Hipertensão arterial sistêmica: proposta de um plano de ação de controle, na Estratégia Saúde da Família, área planalto, nova Mamoré/RO	ODS 3	Nova Mamoré/RO	2	Concluído
Viva bem Itapuã - saúde sim, diabetes e hipertensão não	ODS 3	Itapuã do Oeste/RO	2	Concluído
Masculinidade versus Saúde	ODS 3	Boa Vista/RR	1	Parcialmente concluído
Promoção à boa qualidade de vida	ODS 3	Cantá/RR	1	Concluído
Atividade vital: HIPERDIA em movimento!	ODS 3	Mucajai/RR	1	Concluído
Redução dos fatores de risco relacionados ao desenvolvimento da HAS	ODS 3	Pacaraima/RR	1	Inconcluído

Na etapa EaD, o produto esperado era a elaboração de um projeto de intervenção (PI) por município contemplado. Foram apresentados 17 PIs (Acre, 5; Amazonas 4, Rondônia, 4; e Roraima, 4), dos quais 14 (82,3%) foram totalmente implementados. Ressalta se que os temas relacionados à hipertensão e diabetes representaram 52,9% dos projetos. A descrição dos projetos de intervenção por estado é apresentada no Quadro 1.

## DISCUSSÃO

Para implementar ações de vigilância em saúde, “é necessário pensar sobre a qualificação dos trabalhadores, condição importante para o desenvolvimento de competências e habilidades”^(8)^. Assim, é fundamental oferecer cursos e outras modalidades de educação em saúde que desenvolvam competências, capacitando os profissionais a promover saúde e alcançar metas^(8)^.

A articulação entre ODS 3 e a área da vigilância em saúde é observada no delineamento de metas convergentes no âmbito nacional. O Brasil é um país que aderiu aos ODS e está comprometido com a Agenda 2030. Para acompanhar os objetivos dessa agenda, o Ministério da Saúde implantou o Comitê Consultivo de Monitoramento e Avaliação do Sistema Único de Saúde. Além disso, visando firmar seu compromisso, reelaborou o Plano de Dant com ações relacionadas aos ODS e alinhadas à vigência da Agenda 2030. Nesse cenário, ações formativas que promovam a compreensão dessa agenda e sua articulação com a vigilância em saúde são fundamentais para engajar os profissionais no cumprimento das metas^(9)^.

O curso analisado adotou a modalidade de ensino EaD. Segundo estudo realizado por Brasil et al. (2023), trata-se de “uma modalidade de aprendizagem que flexibiliza a noção de tempo-espaço, favorece o aumento de número de participantes na ação educacional sem perda de qualidade”. Essa modalidade de formação pode ampliar o acesso de profissionais que vivem em áreas remotas e enfrentam dificuldades com deslocamento e afastamento do trabalho para participar de cursos^(10)^.

Os dados relativos aos trabalhadores deste estudo corroboram os achados de uma pesquisa realizada com trabalhadores do Ministério da Saúde, dos quais 67,6% eram do sexo feminino, 53,3% autodeclararam-se brancos, 66,7% tinham pós-graduação, e 38,7% relataram renda entre cinco e dez salários mínimos. Observa-se a predominância do gênero feminino. No nosso estudo, a maioria declarou-se de cor parda, ao passo que, na pesquisa citada, a maioria declarou-se branca. No entanto, note que a região estudada foi a da Amazônia, onde, segundo dados do Instituto Brasileiro de Geografia e Estatística (IBGE), a população é formada majoritariamente por pardos e indígenas. No estudo, a maioria tinha ensino superior completo, enquanto, nos resultados de Matielo et al. (2023), a maior parte dos trabalhadores de saúde eram pós-graduados, remetendo também à área geográfica, já que há menor oferta de cursos de pós-graduação na Região Norte do que nas demais regiões do país^(11,12)^.

Um estudo realizado para avaliar o perfil de atuação de profissionais de vigilância em saúde no estado de Pernambuco mostrou haver uma variação considerável de categorias de profissionais de nível superior em exercício nessa área. Esses dados são confirmados pela nossa pesquisa, a qual identificou psicólogos, farmacêuticos, biomédicos, técnicos e agentes de endemias atuando em vigilância em saúde, apesar do predomínio dos profissionais de enfermagem^(13)^. Mendes et al. (2022) mostraram que o trabalho da enfermagem corresponde a aproximadamente 70% da força de trabalho em saúde no Brasil, porém há desigualdades regionais em sua distribuição: o Sudeste do país tem a “maior concentração desses profissionais, enquanto o Norte e o Nordeste são os mais prejudicados pela distribuição desigual”^(14)^.

Acerca do vínculo de trabalho dos participantes do curso, a maioria (54,8%) tinha contrato temporário, atuando em cargos comissionados ou por meio de vínculo celetista com empresas prestadoras de serviços para o sistema de saúde. Esse aspecto pode favorecer a rotatividade de profissionais da vigilância em saúde, possibilitando uma consequente fragmentação do trabalho e descontinuidade das ações planejadas. Problemas relacionados à rotatividade nesse setor foram relatados por Costa et al (2023): além dos aspectos já mencionados, é destacado o sentimento de não pertencimento à equipe de trabalho e interferências político-partidárias que acabam por desmobilizar as ações de vigilância, prejudicar a dinâmica de execução das atividades e dificultar a articulação da equipe, cujos profissionais estão a todo tempo sendo substituídos por outros^(13,15)^.

Durante o curso, foram elaborados 23 projetos de intervenção, dos quais 16 foram totalmente concluídos. É importante destacar que o diagnóstico situacional de cada município foi analisado previamente para que os participantes identificassem um problema de saúde com base no perfil epidemiológico e no Plano de Dant e, assim, propusessem uma intervenção. Segundo Gallo (2012), as práticas educativas em saúde que consideram o território e sua dinâmica são mais efetivas^(16)^. As Tecnologias da Informação e Comunicação em Saúde (TICS) foram incorporadas à modalidade EaD, superando barreiras geográficas e permitindo aos tutores e participantes do curso receber suporte acadêmico em tempo oportuno. Além disso, as TICS “aumentam a interatividade entre os alunos e tutores e ampliam a perspectiva da produção de conhecimento”^(17)^. Esse suporte pode ter contribuído para que 66,9% dos projetos tenham sido executados até a finalização do curso.

A temática mais abordada (52,9%) nos PIs relacionou-se a hipertensão e diabetes. A escolha dessa temática converge com o estudo que avaliou as mudanças na prevalência e distribuição das DCNT, em que a vulnerabilidade social, as desigualdades e más condições econômicas estão relacionadas ao aumento dessas doenças. A abordagem dessas doenças está diretamente relacionada ao trabalho da enfermagem na atenção primária e vigilância em saúde, além de terem indicadores e metas previstas no Plano de Dant e no ODS 3^(3,18)^.

O trabalho de Melo et al. (2019) identificou uma prevalência de 56,7% das seguintes DCNT: hipertensão, diabetes e dislipidemia. Esse dado foi constatado em uma população de baixa renda e de menor escolaridade, semelhante à de muitos municípios amazônicos, incluindo os abordados neste estudo. A escolha dessas DCNT pela maioria dos participantes pode ter sido motivada pela percepção do recente aumento da sua prevalência nas comunidades locais^(19)^.

A enfermagem tem potencial para contribuir ativa e significativamente para o alcance dos ODS e implementação da Agenda 2030, “começando com uma maior consciencialização através da educação e um compromisso com a investigação e participação na tomada de decisões locais e globais”^(20)^. A força de trabalho da enfermagem é fundamental para o ODS 3; no entanto, o desenvolvimento da liderança, o fortalecimento dos processos educacionais e a pesquisa são requisitos e desafios para que a área da enfermagem, especialmente na América Latina, avance com suas contribuições para o alcance dos ODS^(21)^.

### Limitações do estudo

Preenchimento incompleto dos formulários pelos participantes, resultando em perda amostral.

### Contribuições para a área da Enfermagem e Saúde Pública

Este estudo contribui ao apresentar conhecimentos sobre ações de educação continuada relacionadas ao Desenvolvimento Sustentável e sua interface com a saúde, mais especificamente na área de vigilância de agravos e doenças crônicas não transmissíveis. Destaca-se a participação de profissionais da enfermagem na liderança de processos de planejamento e execução de atividades na vigilância em saúde nos municípios da Amazônia Ocidental brasileira.

## CONCLUSÕES

O curso, ofertado como uma das metas do projeto de extensão que abordou os ODS, a Agenda 2030 e sua interface com Plano de Dant, foi concluído integralmente por 44,08% dos participantes matriculados nos quatro estados da Amazônia Ocidental. Foram desenvolvidos 17 projetos de intervenção com foco nos ODS 3 (Saúde e bem-estar) e 5 (Igualdade de gênero), definidos com base nas prioridades locais e no perfil epidemiológico dos municípios, principalmente no tocante à hipertensão e diabetes *mellitus*. A análise da formação dos trabalhadores da vigilância em saúde revelou que 73,8% tinham nível superior, sendo a enfermagem a categoria profissional mais representativa, o que reforça a importância de seu empoderamento e liderança na implementação da Agenda 2030.

## Data Availability

Os dados da pesquisa estão disponíveis no corpo do artigo.
